# Supplementary material for: Learning earthquake ground motions via conditional generative modeling
Source: Nat Commun. 2026 Mar 16;17:4021. doi: 10.1038/s41467-026-70719-2 (PMC13136394; doi:10.1038/s41467-026-70719-2)
Supplement: Supplementary file 1 — Supplementary information [file 41467_2026_70719_MOESM1_ESM.pdf]

Supplementary information

Contents

1 Background **1**
- 1.1 Details of conditional variables . . . 3
- 1.2 Comparisons of generative models . . . 3

2 Supplementary results **4**
- 2.1 Waveforms showing moderate performance . . . 4
- 2.2 Generated waveforms for H2 component . . . 5
- 2.3 Uncertainty analysis of generated waveforms . . . 5
- 2.4 FAS evaluations across varying earthquake depths . . . 6
- 2.5 FAS evaluations for H2 component . . . 7
- 2.6 FAS maps . . . 8
- 2.7 Evaluations of goodness-of-fit . . . 10
- 2.8 Limited seismic records . . . 11
- 2.9 Residuals computed for the non-ergodic GMM of California . . . 12
- 2.10 Details of fine-tuning and GMM comparisons . . . 13
- 2.11 Spatial correlations . . . 15

This supplementary document provides additional background on generative models, illustrative examples, and a detailed discussion of technical challenges related to our conditional generative modeling approach for earthquake ground motions.

1 Background

This section introduces the technical details of VAE models and their corresponding optimization strategy. VAE [1, 2] has emerged as a powerful framework that bridges probabilistic modeling and deep learning architectures. It has been widely used in image and audio processing to represent complex and high-dimensional data through a learned low-dimensional latent space [3]. As a preliminary step, we first introduce the autoencoder (AE) [4] to provide a clear understanding of its underlying schemes. The general idea is to train a deep neural network to reconstruct the input variable $\mathbf{x} \in \mathbb{R}^N$ with an output variable $\hat{\mathbf{x}} \in \mathbb{R}^N$, where we aim to have $\mathbf{x} \approx \hat{\mathbf{x}}$. An AE architecture, typically shown as a diabolo shape, consists of an encoder and a decoder. Specifically, the encoder module learns a low-dimensional latent representation $\mathbf{z} \in \mathbb{R}^l$ $(l < N)$ of the input data $\mathbf{x}$. The decoder part aims to reconstruct a high-dimensional output $\hat{\mathbf{x}}$ from the low-dimensional feature $\mathbf{z}$.

Furthermore, VAE is an AE model in a probabilistic formulation. The characteristic of VAE lies in that the output from the decoder is a probability distribution of input data $\mathbf{x}$ instead of a deterministic output variable. The encoding of the latent variable $\mathbf{z}$ follows the same probabilistic process, where $\mathbf{z}$ is then a continuous random variable. To be more concrete, the generative process can be defined as

$$\begin{aligned} p_{\boldsymbol{\theta}}(\mathbf{x}) &= \int_{\mathbf{z}} p_{\boldsymbol{\theta}}(\mathbf{x}, \mathbf{z}) d\mathbf{z} \\ &= \int_{\mathbf{z}} p_{\boldsymbol{\theta}}(\mathbf{x}|\mathbf{z}) p_{\boldsymbol{\theta}}(\mathbf{z}) d\mathbf{z}, \end{aligned} \tag{1}$$

where $\boldsymbol{\theta}$ denotes the distribution parameters that are composed of the network weights of the decoder. $p_{\boldsymbol{\theta}}(\mathbf{z})$ represents the model prior over the latent variable $\mathbf{z}$, which is commonly built as an isotropic Gaussian distribution $\mathcal{N}(\mathbf{z}; \mathbf{0}_l, \mathbf{I}_l)$ [1]. $\mathbf{0}_l$ and $\mathbf{I}_l$ are a zero-vector (size l) and an identity matrix (with size l), respectively. Moreover, the likelihood distribution $p_{\boldsymbol{\theta}}(\mathbf{x}|\mathbf{z})$ serves as a probabilistic decoder that generates the observed data $\mathbf{x}$ based on the latent variable $\mathbf{z}$. Generally, let us consider $p_{\boldsymbol{\theta}}(\mathbf{x}|\mathbf{z})$ as a multivariate Gaussian distribution,

$$p_{\boldsymbol{\theta}}(\mathbf{x}|\mathbf{z}) = \mathcal{N}[\mathbf{x}; \boldsymbol{\mu}_{\boldsymbol{\theta}}(\mathbf{z}), \boldsymbol{\sigma}^2_{\boldsymbol{\theta}}(\mathbf{z})], \tag{2}$$

where the mean $\boldsymbol{\mu}_{\boldsymbol{\theta}}(\mathbf{z}) \in \mathbb{R}^N$ and the standard deviation $\boldsymbol{\sigma}_{\boldsymbol{\theta}}(\mathbf{z}) \in \mathbb{R}^N$ are the outputs of the decoding network. Note that the vector $\boldsymbol{\sigma}_{\boldsymbol{\theta}}(\mathbf{z})$ comprises the diagonal coefficients of a diagonal covariance matrix. A diagonal covariance matrix is preferable for computational efficiency [5, 6] due to the quadratic expansion of covariance parameters with respect to (w.r.t.) the variable dimension.

Furthermore, since the space of z in Eq. (1) is large, an approximate posterior $q_{\boldsymbol{\phi}}(\mathbf{z}|\mathbf{x})$ is used to reduce the computational effort. $q_{\boldsymbol{\phi}}(\mathbf{z}|\mathbf{x})$ works as a probabilistic encoder that is parameterized by $\boldsymbol{\phi}$. It is formulated as

$$q_{\boldsymbol{\phi}}(\mathbf{z}|\mathbf{x}) = \mathcal{N}[\mathbf{z}; \boldsymbol{\mu}_{\boldsymbol{\phi}}(\mathbf{x}), \boldsymbol{\sigma}^2_{\boldsymbol{\phi}}(\mathbf{x})]. \tag{3}$$

Here $\boldsymbol{\mu}_{\boldsymbol{\phi}}(\mathbf{x}) \in \mathbb{R}^l$ and $\boldsymbol{\sigma}_{\boldsymbol{\phi}}(\mathbf{x}) \in \mathbb{R}^l$ are the outputs of the encoder w.r.t. the data $\mathbf{x}$. Similarly, $\boldsymbol{\sigma}_{\boldsymbol{\phi}}(\mathbf{x})$ consists of the diagonal elements in a diagonal covariance matrix.

The training of a VAE involves optimizing the evidence lower bound (ELBO) on the marginal likelihood of the observed data $\mathbf{x}$. For a given data sample $\mathbf{x}$, the marginal likelihood is defined as

$$\begin{aligned} \log p_{\boldsymbol{\theta}}(\mathbf{x}) &= \mathrm{D}_{\mathrm{KL}}(q_{\boldsymbol{\phi}}(\mathbf{z}|\mathbf{x})||p_{\boldsymbol{\theta}}(\mathbf{z}|\mathbf{x})) + \mathcal{L}(\boldsymbol{\theta}, \boldsymbol{\phi}; \mathbf{x}) \\ &\geqslant \mathcal{L}(\boldsymbol{\theta}, \boldsymbol{\phi}; \mathbf{x}), \end{aligned} \tag{4}$$

where $\mathrm{D}_{\mathrm{KL}}(\cdot)$ denotes the Kullback-Leibler (KL) divergence between the approximate and true posterior distributions. $\mathrm{D}_{\mathrm{KL}}(\cdot)$ is a non-negative term. Therefore, $\mathcal{L}(\boldsymbol{\theta}, \boldsymbol{\phi}; \mathbf{x})$ represents the ELBO, which is given by [1]

$$\mathcal{L}(\boldsymbol{\theta}, \boldsymbol{\phi}; \mathbf{x}) = \mathbb{E}_{q_{\boldsymbol{\phi}}(\mathbf{z}|\mathbf{x})}[\log p_{\boldsymbol{\theta}}(\mathbf{x}|\mathbf{z})] - \mathrm{D}_{\mathrm{KL}}(q_{\boldsymbol{\phi}}(\mathbf{z}|\mathbf{x})||p_{\boldsymbol{\theta}}(\mathbf{z})). \tag{5}$$

The first and second terms on the right-hand side (RHS) are reconstruction loss and a KL-divergence term, respectively. The KL-divergence works as a regularizer for $\boldsymbol{\phi}$ that promotes the approximate posterior $q_{\boldsymbol{\phi}}(\mathbf{z}|\mathbf{x})$ to closely resemble the prior $p_{\boldsymbol{\theta}}(\mathbf{z})$. Note that the KL-divergence term in (5) is analytically tractable but the reconstruction error term requires estimation via Monte Carlo sampling. Thus, the expectation w.r.t. $q_{\boldsymbol{\phi}}(\mathbf{z}|\mathbf{x})$ can be estimated by

$$\mathbb{E}_{q_{\boldsymbol{\phi}}(\mathbf{z}|\mathbf{x})}[\log p_{\boldsymbol{\theta}}(\mathbf{x}|\mathbf{z})] \approx \frac{1}{R}\sum_{r=1}^{R} \log p_{\boldsymbol{\theta}}(\mathbf{x}|\mathbf{z}^{(r)}), \tag{6}$$

where R samples $\mathbf{z}^{(r)}$ are independently and identically drawn from $q_{\boldsymbol{\phi}}(\mathbf{z}|\mathbf{x})$. Given a dataset $\mathbf{X} = \{\mathbf{x}_i\}_{i=1}^M$, where $\mathbf{X}$ consists of M independent and identically distributed (i.i.d.) samples, the resulting estimator of ELBO is written as,

$$\begin{aligned} \mathcal{L}(\boldsymbol{\theta}, \boldsymbol{\phi}; \mathbf{X}) = &\sum_{i=1}^{M} \log p_{\boldsymbol{\theta}}(\mathbf{x}_i|\mathbf{z}_i) \\ &- \sum_{i=1}^{M} D_{KL}(q_{\boldsymbol{\phi}}(\mathbf{z}_i|\mathbf{x}_i)||p_{\boldsymbol{\theta}}(\mathbf{z}_i)). \end{aligned} \tag{7}$$

To optimize (7), stochastic gradient descent (SGD) methods, such as Adam [7], are typically considered. The ELBO $\mathcal{L}(\boldsymbol{\theta}, \boldsymbol{\phi}; \mathbf{X})$ is optimized w.r.t. both the generative parameters $\boldsymbol{\theta}$ and the variational parameters $\boldsymbol{\phi}$. Note that $\mathcal{L}(\boldsymbol{\theta}, \boldsymbol{\phi}; \mathbf{X})$ is differentiable w.r.t $\boldsymbol{\theta}$ but it is problematic to obtain the derivatives w.r.t. $\boldsymbol{\phi}$. To solve this issue, the reparameterization trick is proposed [1] to conduct a differentiable transform, where $\mathbf{z}_i$ can be reparameterized as

$$\mathbf{z}_i \sim \mathcal{N}(\boldsymbol{\mu}_{\boldsymbol{\phi}}(\mathbf{x}_i), \boldsymbol{\sigma}^2_{\boldsymbol{\phi}}(\mathbf{x}_i)), \tag{8}$$

where $\boldsymbol{\sigma}_{\boldsymbol{\phi}}(\mathbf{x}_i)$ comprises the diagonal elements in a diagonal covariance matrix.

1.1 Details of conditional variables

The embedding module incorporates the 3D setup of geospatial coordinates, including longitude, latitude, and depth of the source and site locations. This allows our CGM-GM model to learn and capture the complex spatiotemporal patterns of earthquake data. To be more concrete, let us consider a general case of predicting seismic waveforms at a station x_r generated from a source x_s, which is given by

$$\mathcal{F}(t, x_s, x_r, m) = \mathcal{S}(t, x_s) * \mathcal{R}(t, x_r) * \mathcal{G}(t, x_s, x_r), \tag{9}$$

where $\mathcal{F}(\cdot)$ denotes the prediction function that generates waveforms. $\mathcal{S}(\cdot)$, $\mathcal{R}(\cdot)$, and $\mathcal{G}(\cdot)$ represent the source effects, site effects, and Green's function (or path effects), respectively. $*$ is the convolution. In our conditional generative modeling framework, this function $\mathcal{F}(\cdot)$ is approximated by the neural networks. Specifically, we incorporate physical variables (i.e., earthquake magnitudes, depths, source and receiver locations) as conditional variables v. In our implementation, x_s represents source parameters, including magnitudes, source locations, and depths. The CGM-GM framework incorporates source effects, such as source mechanisms and their coupling with near-source geology. Note that this leads to implicitly assuming a specific source mechanism at each source location, which is reasonable. The conditional variable of a receiver location corresponds to x_r and we incorporate the site effect $\mathcal{R}(x_r, t)$. We do not separately model $\mathcal{S}(\cdot)$, $\mathcal{R}(\cdot)$ and $\mathcal{G}(\cdot)$, and hence these effects are learned as a combination through the neural network. We interpret that using many earthquake events allows us to learn $\mathcal{R}(\cdot)$ at a specific location, and using many stations lets us learn $\mathcal{S}(\cdot)$ for a specific event. Similarly, by using many earthquake-station pairs, we can learn Green's function $\mathcal{G}(\cdot)$.

1.2 Comparisons of generative models

The primary focus of our work is to generate spatially continuous waveforms and demonstrate the capability of generative AI methods to learn the underlying wave propagation characteristics and spatial heterogeneity in ground motion data. Although the choice of generative model is important, it remains a secondary consideration in this study. We select the dynamic VAE model for its lightweight architecture, stable training process, and computational efficiency, which provide a robust foundation to address the specific challenges of our research. More broadly, we aim to make this framework accessible to researchers and domain scientists who may not have extensive experience with generative modeling methods. Meanwhile, we acknowledge the potential of alternative approaches, such as generative adversarial networks (GANs) [8] and diffusion models (DMs) [9, 10].

Each generative model has distinct strengths and limitations, which makes their choice dependent on specific applications and research goals. Supplementary Table 1 provides an overall comparison of commonly used generative models across four crucial aspects: generation fidelity, sampling efficiency, training stability, and compression [11]. In addition to the previously mentioned benefits

Supplementary Table 1: An overview of generative models in terms of generation fidelity, sampling efficiency, training stability, and compression (✓: Good, ✗: Suboptimal).

Model	High fidelity	Fast sampling	Training stability	Compression
VAE	✗	✓	✓	✓
GAN	✓	✓	✗	✗
DM	✓	✗	✓	✗

of VAE models, another advantage is their ability to perform compression, enabling the learning of compact representations of complex data. While VAE models may not yet achieve the same level of fidelity as other generative methods (GANs and DMs), we hope this work inspires future research aimed at further improving generative modeling frameworks for synthesizing spatially continuous ground motion waveforms [12].

GANs are widely recognized for their capability to generate high-fidelity samples. However, their training process is often prone to instability, which can pose challenges in practice [13]. Furthermore, GANs do not provide a density function, presenting the direct estimation of the probability of a sample from the true underlying data distribution [11, 14]. Despite these limitations, GANs have achieved success in various applications and remain a competitive choice for generative modeling. Recently, DMs have emerged as a powerful alternative, offering both high fidelity and training stability. However, their effectiveness often relies on large training datasets and substantial computational resources, making them less practical in data-constrained scenarios. Moreover, DMs often require tens to thousands of time discretization steps of the learned diffusion process to achieve the desired accuracy, leading to relatively slow sampling processes. Numerous advancements have been proposed to address these challenges [15], and ongoing research continues to focus on improving their efficiency. DMs represent a promising direction for future research, and we have begun exploring their potential in follow-up work [12].

2 Supplementary results

In this section, we present supplementary generated samples to evaluate the performance of our CGM-GM framework in terms of waveform shapes, uncertainty quantification, and FAS evaluations. For a comprehensive analysis, we provide generative results for both the H1 and H2 components. Additionally, we set up two scenarios to test the realization of FAS maps in consideration of site and source effects.

2.1 Waveforms showing moderate performance

The generative process exhibits inherent stochasticity, and some of the generated ground motions demonstrate moderate performance when compared to the actual ground motions. Based on the H1 components, we conduct 100 generations for each set of conditional variables and select waveforms that exhibit moderate performance for presentation in Supplementary Figure 1. This figure showcases a variety of waveforms corresponding to different earthquake magnitudes, rupture distances, and earthquake depths. Specifically, we observe unrealistic spikes in some waveform shapes, which resemble audio signals as indicated by the black boxes in Supplementary Figure 1. These artifacts are attributed to the use of phase retrieval methods, which may result in inaccurate phase information and consequently distorted waveform shapes. Additionally, numerical errors introduced by phase retrieval methods may lead to the mismatch of arrival times, as illustrated in

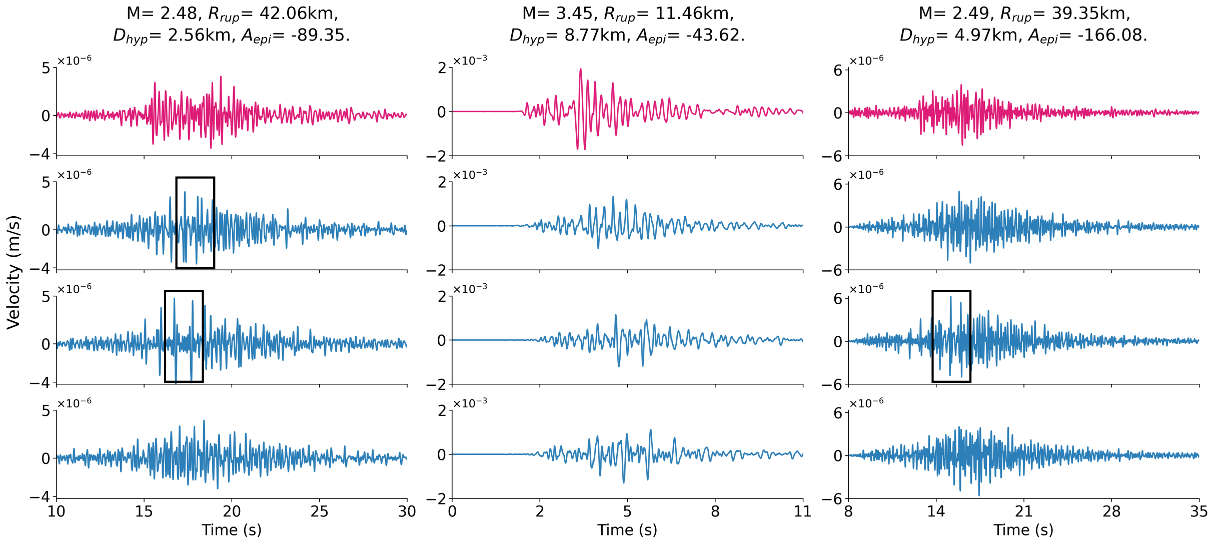

Supplementary Figure 1: Illustrative generated waveforms showing moderate performance. For each scenario, three waveforms are randomly generated given the same conditional variables. The first row presents the ground truth data (red colored) while the rest of them show the corresponding generations (blue colored). The black boxes show the spike-like issues in the generated waveforms. Note that the waveforms are displayed based on their individual motion durations.

the middle column of Supplementary Figure 1. To address this potential issue, a promising solution would be to learn the waveforms directly in the time domain instead of the time-frequency domain to avoid using phase retrieval methods. Moreover, since VAE models tend to produce relatively smooth amplitude information, which may introduce numerical artifacts during phase retrieval, the quality of generated waveforms could be improved by employing alternative generative frameworks such as diffusion models in follow-up work [12].

2.2 Generated waveforms for H2 component

The H2 component denotes the recorded waveforms from the North-South (N-S) direction. We also trained a generative model using the H2 component, which produces consistent waveforms across various pairs of conditional variables. As illustrated in Supplementary Figure 2, our CGM-GM approach effectively captures realistic waveform shapes, peak velocity values, and arrival times. This further validates the robustness and applicability of our framework to diverse ground motion datasets.

2.3 Uncertainty analysis of generated waveforms

Supplementary Figure 3 illustrates the uncertainty of generations based on 100 simulated samples. Multiple pairs of physical parameters are considered for a comprehensive investigation. The mean curves of the generated data (blue) generally capture the dynamic patterns of ground motion waveforms, though they do not precisely match the true recordings. The blue-shaded regions, representing the predicted mean ± one standard deviation (std), demonstrate good coverage of the ground truth data (red).

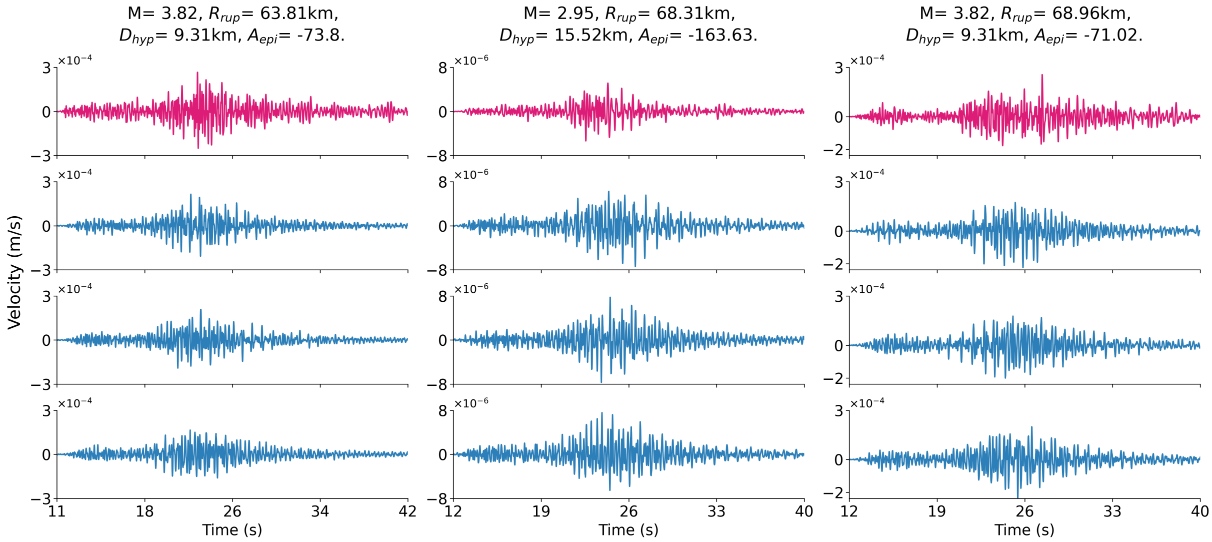

Supplementary Figure 2: Illustrative generated waveforms for H2 component. For each scenario, three waveforms are randomly generated given the same conditional variables. The first row presents the ground truth data (red) while the rest of them show the corresponding generations (blue). Note that the waveforms are displayed based on their individual motion durations.

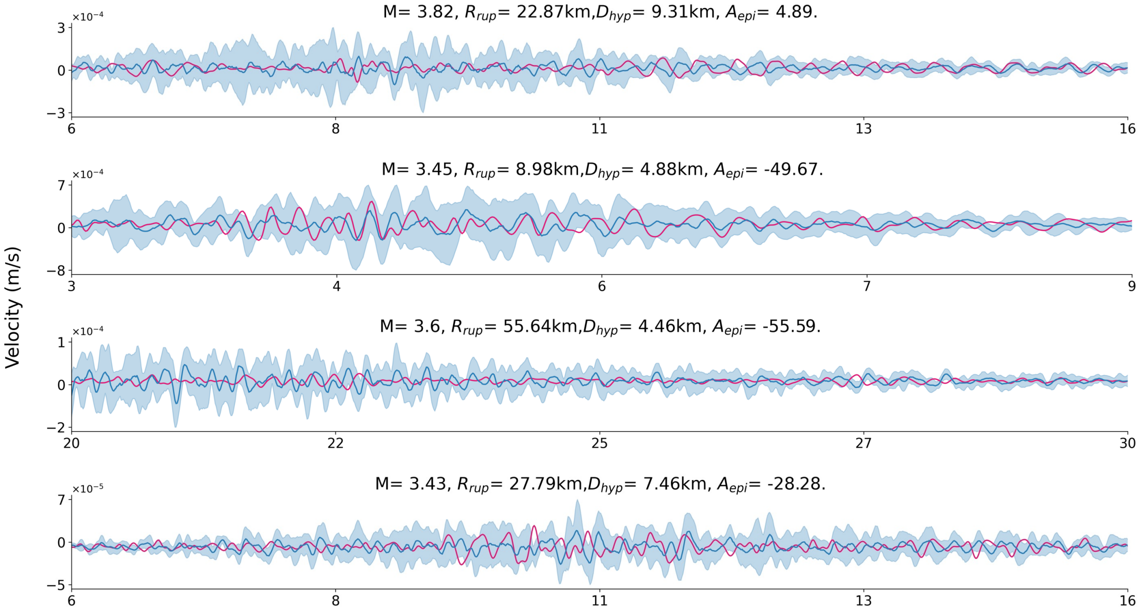

Supplementary Figure 3: Illustrative examples of the uncertainty of generated samples. The red and blue curves represent the true and predicted mean waveforms, respectively. The blue shading regions denote the coverages of the mean ± one std. Note that the waveforms are displayed based on their individual motion durations.

2.4 FAS evaluations across varying earthquake depths

Moreover, we show the FAS results across varying earthquake depths. Earthquake depths play a significant role in FAS values by affecting the attenuation, wave propagation path, site effects, source radiation pattern, and others. In this part, we generate 100 waveforms for each set of conditional variables from the H1 component as a showcase and calculate the corresponding FAS values for

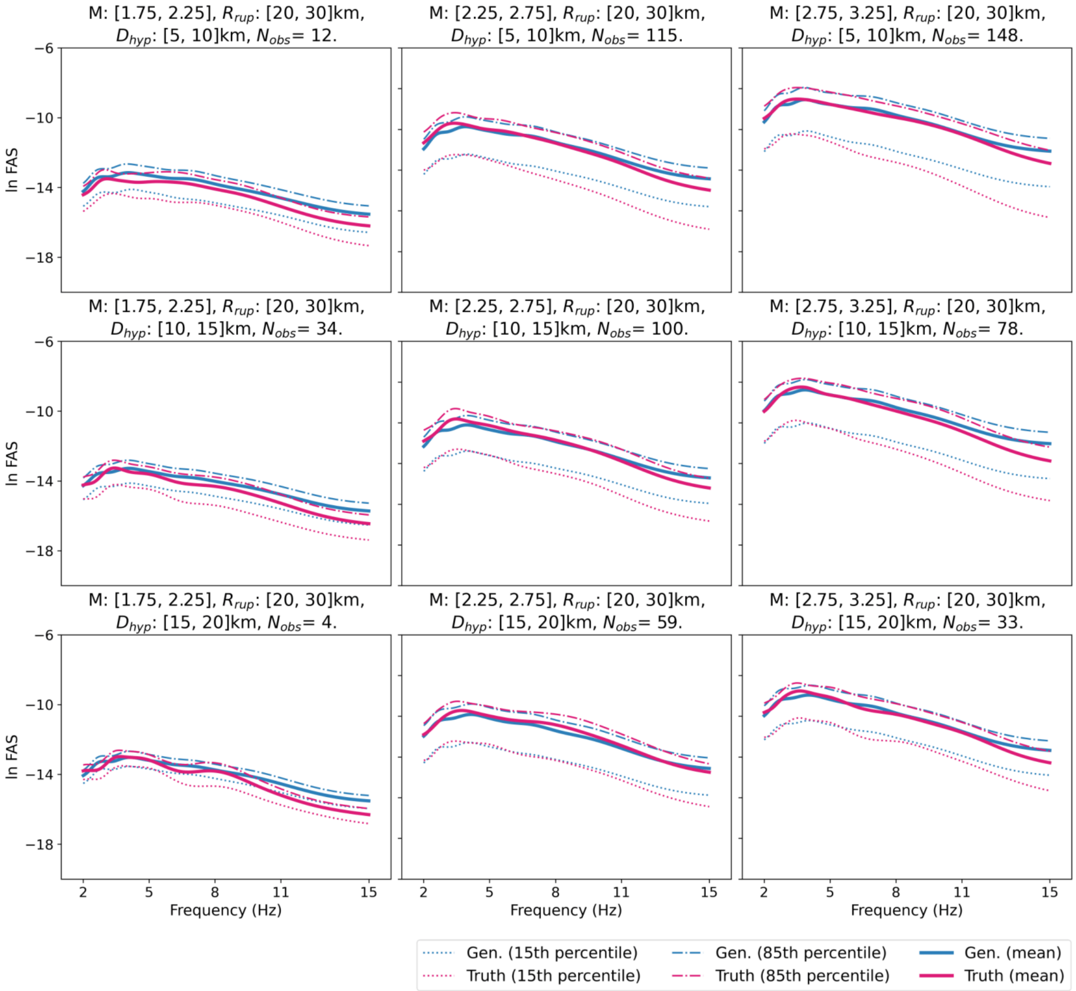

Supplementary Figure 4: Fourier Amplitude Spectra (FAS) comparisons with varying earthquake depths for the H1 component between the ground truth (red) and the generations (blue). The rupture distances are within a fixed range. The FAS results are presented at the 15th percentile, mean, and 85th percentile. "Gen." denotes the results from generations.

quantitative analysis. As exhibited in Supplementary Figure 4, with the rupture distance R_{rup} fixed, we present the FAS comparisons along earthquake magnitudes M (rows) and the earthquake depths D_{hyp} (columns). Overall, the generated results align well with the true FAS values, although slight mismatches are observed in the high-frequency regions. Another interesting finding is that the generated ground motion data demonstrates a closer agreement with the ground truth FAS values as earthquake depth increases. This improved performance is likely due to reduced attenuation, which facilitates more effective learning of the generative models.

2.5 FAS evaluations for H2 component

Supplementary Figures 5 and 6 illustrate FAS comparisons for the H2 component across various rupture distances (R_{rup}) and earthquake depths (D_{hyp}), respectively. Overall, the generated FAS results match the true FAS values well. In Supplementary Figure 5, as rupture distances increase (see each column), the predicted FAS values exhibit more discrepancies compared to the true recordings. In Supplementary Figure 6, similar to our observations with the H1 component, larger earthquake

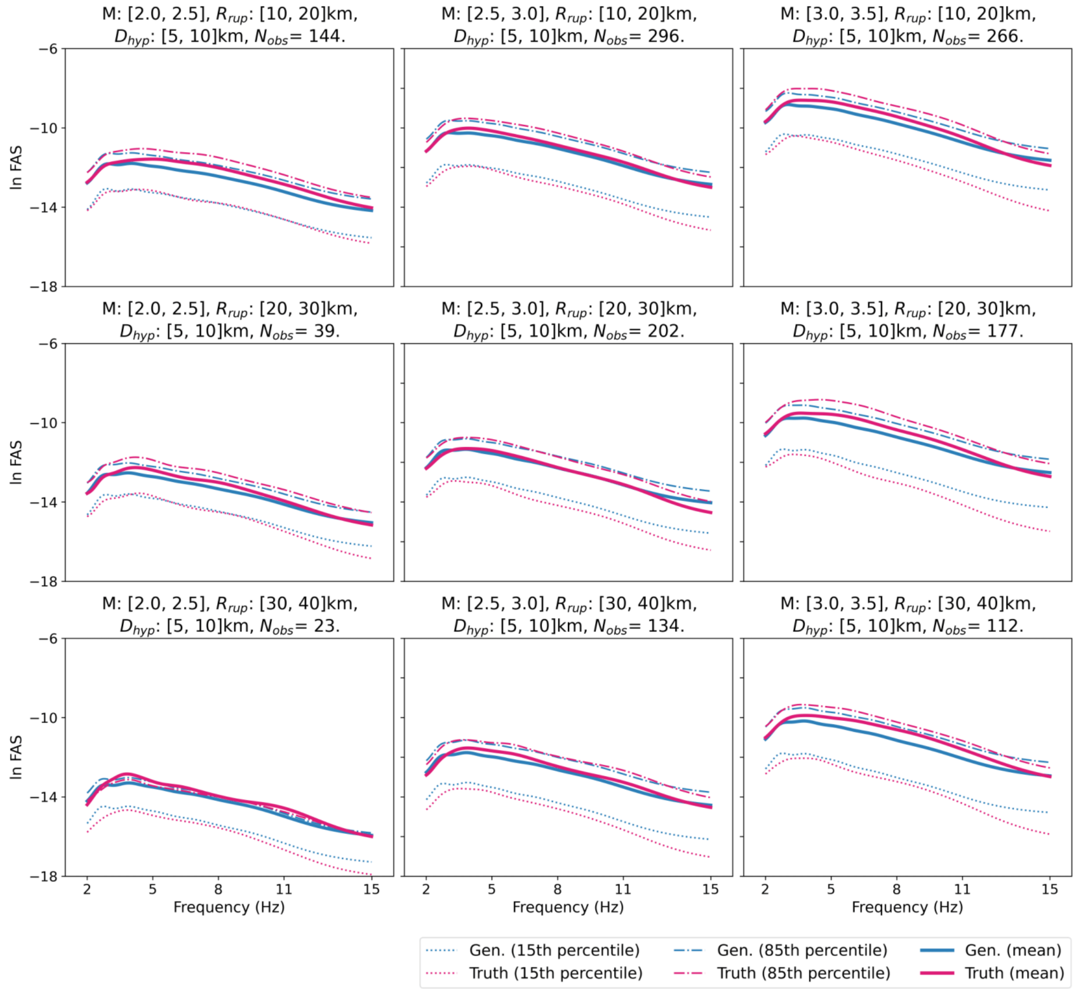

Supplementary Figure 5: Fourier Amplitude Spectra (FAS) comparisons with varying rupture distances for the H2 component between the ground truth (red) and the generations (blue). The earthquake depths are within a fixed range. The FAS results are presented at the 15th percentile, mean, and 85th percentile. "Gen." denotes the results from generations.

depths result in better agreement between the generated FAS and the ground truth. The FAS analysis on the H2 component also demonstrates the feasibility of our method for producing realistic ground motion waveforms, especially for engineering applications.

2.6 FAS maps

In this part, we provide the comparative results of FAS maps from CGM-GM, ergodic GMM, and non-ergodic GMM. Note that the generated FAS maps from CGM-GM represent the median across 30 independent realizations. Firstly, apart from the comparison of FAS maps at 10 Hz in the main text, we also show the results at a frequency of 5 Hz and 2 Hz in Supplementary Figures 7 and 8, resepctively. The earthquake source is located at a latitude and longitude of $(37.86^\circ, -122.26^\circ)$ and a depth of 7.94 km. The earthquake magnitude is 3.84. Moreover, we present supplementary FAS simulation results by manually designing two scenarios: (a) a single epicenter with multiple seismic stations and (b) a single seismic station with multiple epicenters. These scenarios evaluate the site and source effects within a selected region Ω in the SFBA, bounded

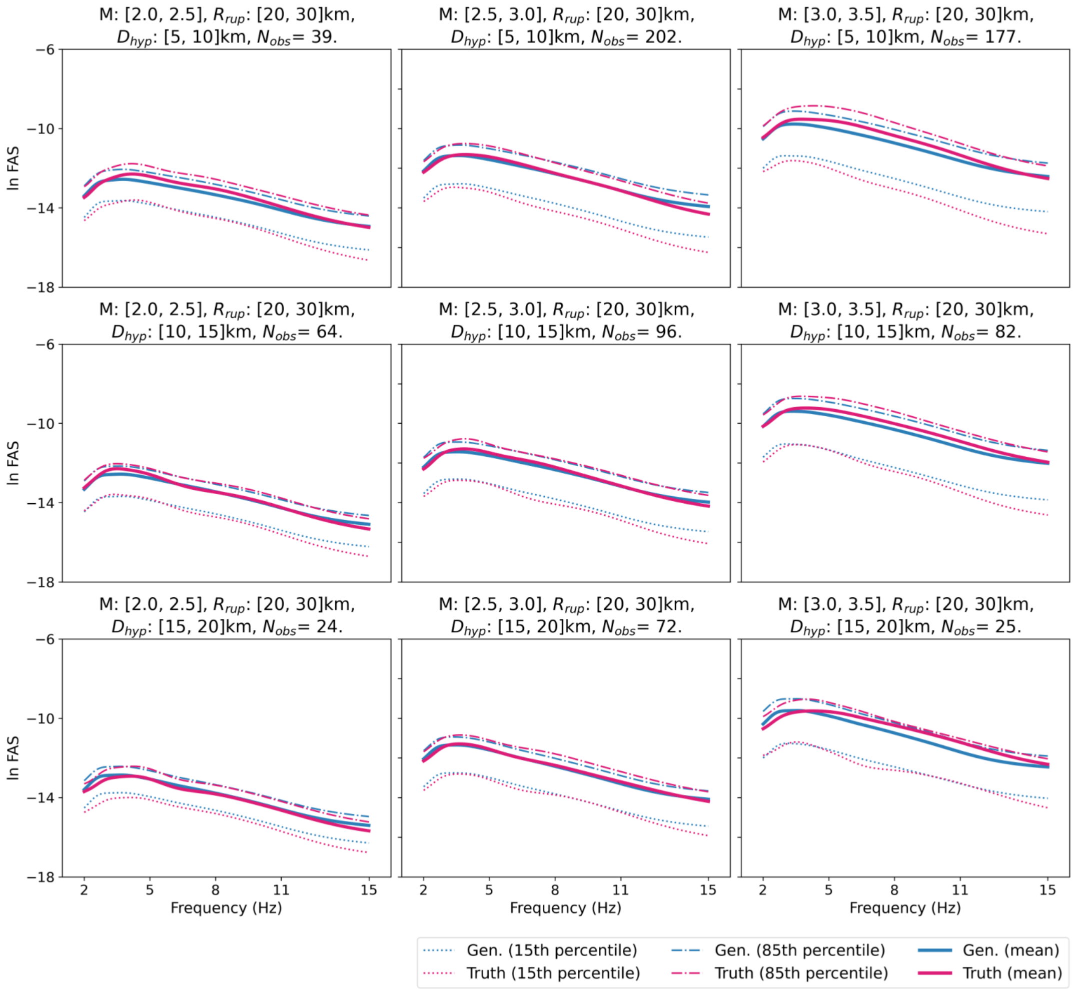

Supplementary Figure 6: Fourier Amplitude Spectra (FAS) comparisons with varying earthquake depths for the H2 component between the ground truth (red) and the generations (blue). The rupture distances are within a fixed range. The FAS results are presented at the 15th percentile, mean, and 85th percentile. "Gen." denotes the results from generations.

by latitudes $[37°15.3'N, 38°03.8'N]$ and longitudes $[121°14.5'W, 121°28.8'W]$. Hence, two FAS maps are generated using our CGM-GM framework and compared against results from empirical ground-motion models (GMMs), including both ergodic and non-ergodic types.

For scenario (a), we select an epicenter along the Hayward fault, which is located at a geographic position with a latitude of $37°28.1'N$ and a longitude of $121°47.5'W$. This seismic event is characterized by a magnitude of 3.0 and an earthquake depth of 5.0 km. The stations are defined as a uniform grid of 100×100 in the spatial domain Ω. By leveraging those conditional variables, we generate 10,000 waveform samples and compute the corresponding FAS values at 10 Hz using our generative model, the ergodic and non-ergodic GMMs, as shown in Supplementary Figure 9(**a**-**c**). The comparative results indicate that the generative model can generally produce meaningful FAS values for spatial interpolation with under-sampled observation data. Note that the unexpected "cross-shaped" pattern in Supplementary Figure 9(**a**) is due to the sparsity of seismic stations across the SFBA region.

For scenario (b), an observation station is chosen at a geographic position with a latitude of

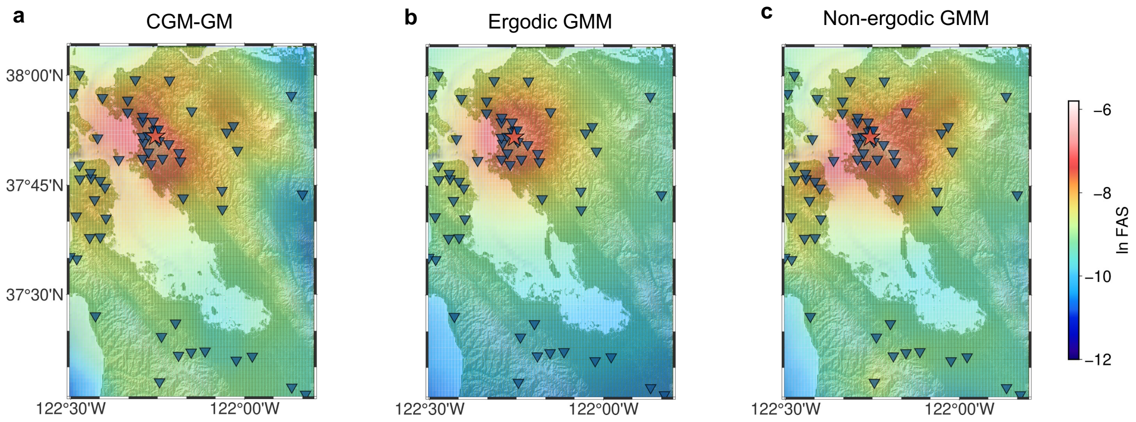

Supplementary Figure 7: Comparative results of Fourier Amplitude Spectra (FAS) maps at a frequency of 5 Hz between our generations and the empirical ground motion models (GMMs). This seismic event is defined with a magnitude of 3.84 and a depth of 7.94 km. The epicenter (red star) is located at a geographic position with a latitude of $37°51.6'N$ and a longitude of $122°15.6'W$. **a**, **b**, and **c** show the FAS maps of our generative model, ergodic GMM, and non-ergodic GMM. Our generated FAS map represents the median across 30 independent realizations.

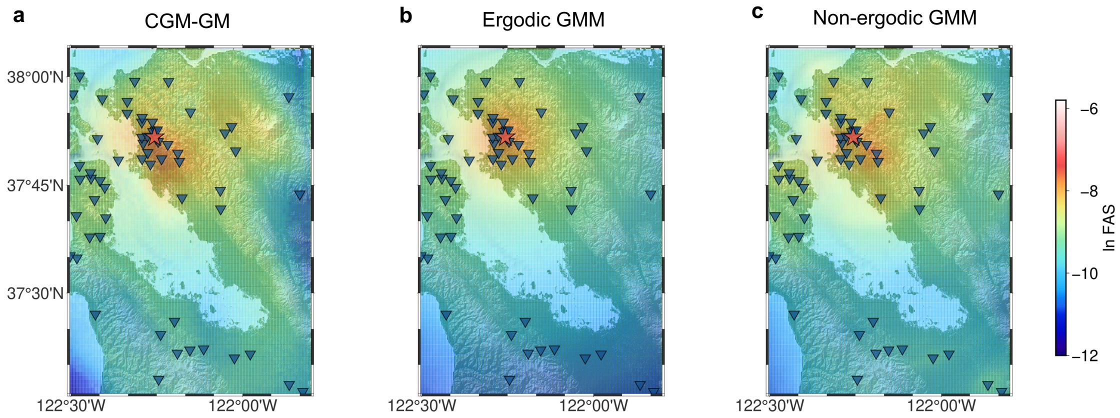

Supplementary Figure 8: Comparative results of Fourier Amplitude Spectra (FAS) maps at a frequency of 2 Hz between our generations and the empirical ground motion models (GMMs). Our generated FAS map represents the median across 30 independent realizations.

$37°44.3'N$ and a longitude of $122°10.9'W$. All the seismic events are defined with a magnitude of 2.48 and a depth of 6.2 km. We sample the epicenters with a uniform grid of 100×100 in Ω. The FAS values are obtained based on these geophysical conditions. Supplementary Figure 10(**a-c**) presents comparative results of our generative model, the ergodic and non-ergodic GMMs. Our generated FAS map also exhibits a good agreement with that from empirical GMMs. Additionally, we do not observe a similar "cross-shaped" pattern as shown in Supplementary Figure 9(**a**) due to a more uniform distribution of earthquake sources in the SFBA dataset. Overall, we validate the effectiveness of our generative modeling pipeline for ground motion simulation, especially for spatial interpolation.

2.7 Evaluations of goodness-of-fit

Additionally, we can evaluate the quality of the generated waveforms using established goodness-of-fit (GoF) metrics for broadband ground motion [16], such as Anderson's criteria [17] and its

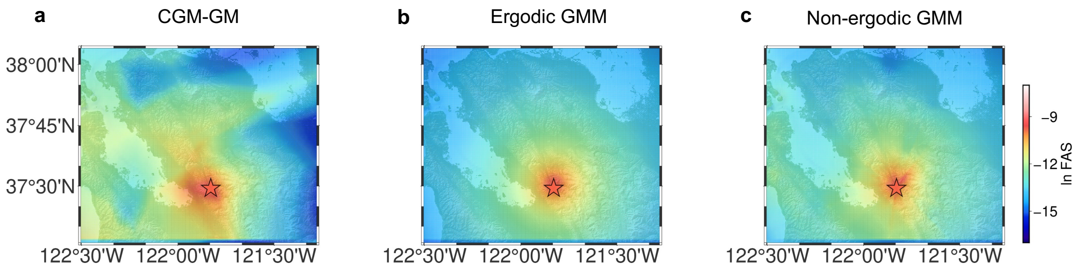

Supplementary Figure 9: Comparative results of Fourier Amplitude Spectra (FAS) maps at a frequency of 10 Hz on the scenario (a) between our generations and the empirical ground motion models (GMMs). This seismic event is manually defined with a magnitude of 3.0 and a depth of 5.0 km. The epicenter (red star) is located at a geographic position with a latitude of $37^{\circ}28.1'N$ and a longitude of $121^{\circ}47.5'W$. **a**, **b**, and **c** show the FAS maps of our generative model, ergodic GMM, and non-ergodic GMM. Our generated FAS map represents the median across 30 independent realizations.

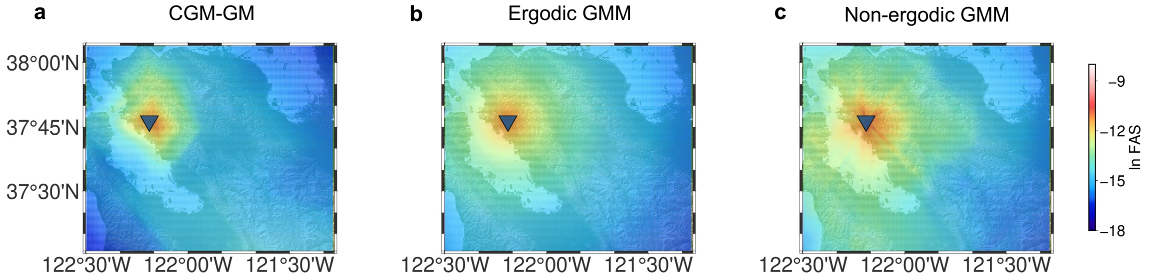

Supplementary Figure 10: Comparative results of Fourier Amplitude Spectra (FAS) maps at a frequency of 10 Hz on the scenario (b) between our generations and the empirical ground motion models (GMMs). The seismic station (blue triangle) is located at a geographic position with a latitude of $37^{\circ}44.3'N$ and a longitude of $122^{\circ}10.9'W$. All the seismic events are defined with a magnitude of 2.48 and a depth of 6.2 km. **a**, **b**, and **c** show the FAS maps of our generative model, ergodic GMM, and non-ergodic GMM. Our generated FAS map represents the median across 30 independent realizations.

modified version proposed by Olsen *et al.* [18]. In this study, we adopt the original Anderson's criteria to assess the GoF of the synthetic waveforms, providing a comprehensive evaluation of our model's capability to generate realistic broadband ground motions.

The Anderson's criteria have ten distinct characteristics, each evaluated on a scale from 0 to 10. A score of 10 indicates perfect agreement. The scores for individual parameters are averaged to determine the overall GoF. A score below 4 denotes a poor fit, 4–6 represents a fair fit, 6–8 indicates a good fit, and scores exceeding 8 are an excellent fit. The GoF analysis has been performed across different rupture distances, earthquake depths, and magnitudes, with results presented in Supplementary Figure 11. Our findings indicate that the generated waveforms exhibit a good agreement with the ground truth, with mean GoF values generally around 6.

2.8 Limited seismic records

Our proposed method is designed for applicability across different regions. To validate its effectiveness, we first introduce the requirements for implementing the CGM-GM framework and then evaluate its performance under a scenario with limited seismic records. The main data components include recorded seismic waveforms and their corresponding conditional variables, i.e., earthquake magnitudes, source depths, and geospatial coordinates of sources and stations. Seismic stations of interest can be selected based on a specific range of magnitudes and rupture distances. The

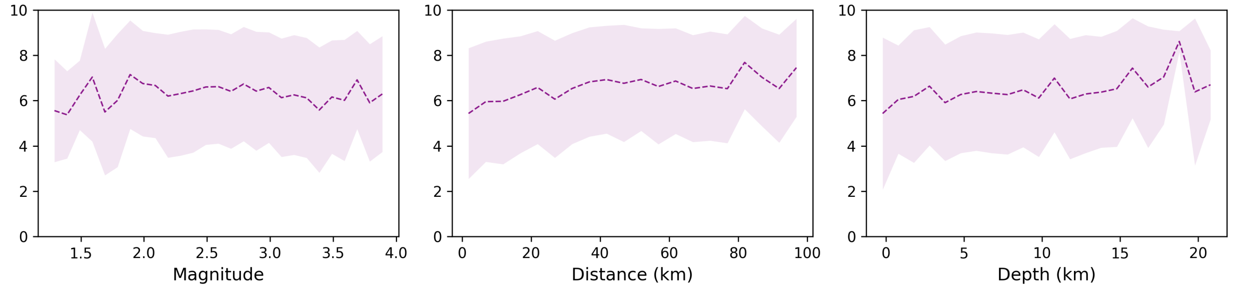

Supplementary Figure 11: An overview of goodness-of-fit (GoF) values between our generations and ground truth observations across various magnitudes, rupture distances R_{rup}, and earthquake depths. The dashed curves and shading regions denote the mean values and the coverages of the mean $\pm$ one std, respectively.

implementation process can be summarized into two parts: (i) preprocessing the datasets and (ii) applying the CGM-GM framework. The preprocessing procedures are described in Section *Data selection*. We perform the Fourier transforms to both the noise and the signal and smooth the Fourier amplitudes using the Konno-Ohmachi [19] window procedure. Our goal is to retain recordings with a signal-to-noise (S/N) ratio exceeding 3 across the frequency range of $[2, 15]$ Hz. To apply the CGM-GM model, we first extract time-frequency amplitude information using the STFT. The dynamic VAE model is then trained on amplitude data alongside the conditional variables. Once the model is well trained, we generate synthetic ground motions by sampling from the prior distribution and conditioning on specific input variables.

To assess the robustness of our CGM-GM model under limited seismic datasets, we evaluate its performance on additional scenarios with reduced station and source coverage. Specifically, we design two cases of data sparsity by independently reducing the number of stations and sources in the original training dataset. We introduce a random removal ratio λ, selected from $[0.01, 0.1, 0.2, 0.3, 0.4, 0.5, 0.9]$, to systematically reduce data availability. For each removal scenario, we train three independent models using three different random seeds, ensuring variability in the selected waveform samples and facilitating a fair comparison. For instance, applying $\lambda = 0.3$ to station removal results in a dataset containing 3880 waveforms for training. The variation in random seeds ensures different subsets of waveforms are removed in each instance. To quantify model performance, we compute the average absolute difference between the predicted and true FAS values across all data samples. The results of this evaluation under reduced data availability are presented in Supplementary Figure 12. Moreover, Supplementary Figure 13 presents a comparison of FAS maps (10 Hz) for the non-ergodic GMM, CGM-GM trained with the full dataset, and CGM-GM trained under data-limited scenarios, where source removal ratios are defined as 0.3, 0.5, and 0.9. The seismic event used for producing the FAS maps is consistent with the event in Figure 2 of the main text, which is characterized by a magnitude of 3.84 and a depth of 7.94 km. The epicenter, denoted by a red star, is located at a geographic position with a latitude of $37°51.6'N$ and a longitude of $122°15.6'W$. Our model demonstrates robust performance, maintaining reasonably good performance and successfully generating realistic FAS maps even with a 30% reduction in data availability. These results validate the effectiveness of our approach in handling limited seismic records.

2.9 Residuals computed for the non-ergodic GMM of California

In addition to the comparisons with the non-ergodic and ergodic GMMs specifically developed for small-magnitude earthquakes in Section Empirical GMMs, we evaluate the performance of CGM-

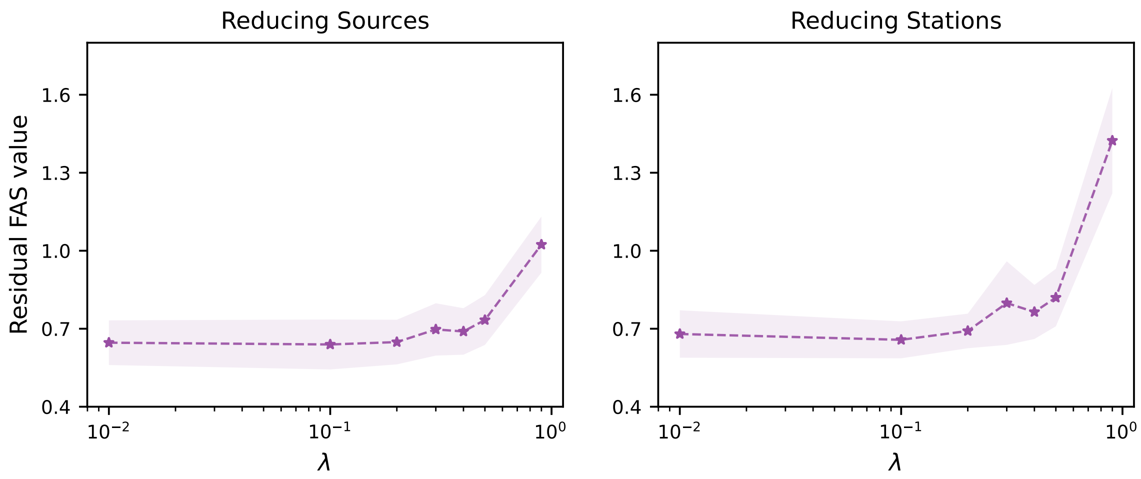

Supplementary Figure 12: Model performance of our CGM-GM on limited seismic records. We reduce the number of earthquake sources and stations in the original dataset. The results are based on three random runs. The purple curves represent the average absolute difference between predicted and true Fourier Amplitude Spectra (FAS) values across all waveform samples. The shading regions denote the coverages of the mean ± one std.

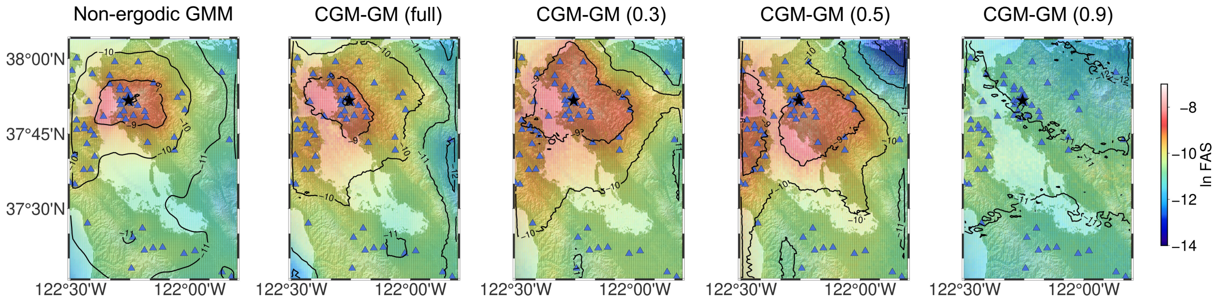

Supplementary Figure 13: A comparison of Fourier Amplitude Spectra (FAS) maps at 10 Hz for the non-ergodic ground motion model (GMM), CGM-GM trained with the full dataset, and CGM-GM trained under data-limited scenarios, where source removal ratios are defined as 0.3, 0.5, and 0.9.

GM against the non-ergodic LA21 model developed for California by [20]. We focus on FAS within the $[2, 15]$ Hz frequency range for events with magnitudes greater than 3, corresponding to the valid range of the LA21 model and yielding to 3,326 traces for evaluation. We compute logarithmic residuals of FAS values between the observed data and the predictions from both CGM-GM and LA21. Note that we use the median predictions from 30 realizations for CGM-GM. Due to limited availability of site-specific parameters (V_{S30} and $Z_{1.0}$) at most stations, we estimate these values based on the USGS San Francisco Bay Region 3D Seismic Velocity Model (Version 21.1) [21]. As shown in Supplementary Figure 14, CGM-GM and LA21 models lead to a similar zero average in the residuals. CGM-GM exhibits a slightly lower residual range, especially at low (< 3 Hz) and high (> 10 Hz) frequencies.

2.10 Details of fine-tuning and GMM comparisons

To compute distances between the fault plane and the stations, we assume finite faults are centered at a latitude of $37°51.6'N$ and a longitude of $122°15.6'W$, which corresponds to the hypocenter location used in small earthquake comparisons. We determine fault widths and lengths based on

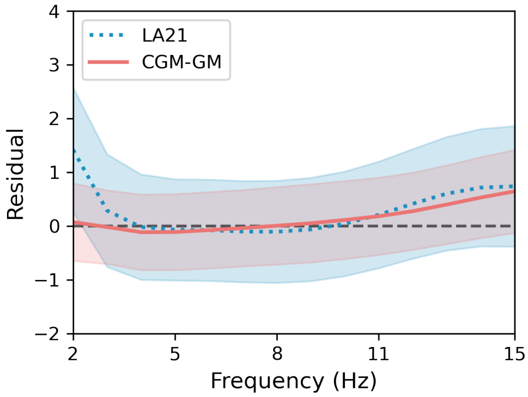

Supplementary Figure 14: The comparison of Fourier Amplitude Spectra (FAS) residuals between the CGM-GM predictions and the LA21 non-ergodic ground motion model (GMM). Residuals are defined as the logarithmic difference between the model predictions and the observed ground truth values. The solid line and the shaded area denote the mean curves and the region of mean ± std.

the fault-scaling relations in [22] (Table 5). For site parameters, we follow the procedure described in 2.9. We then calculate FAS predictions at 10 Hz for these site conditions and take the mean values. Stations within rupture distance R_{rup} of 10 to 50 km are selected for this analysis.

In Figure 5 of the main text, we present comparisons for rupture distances ranging from 10 to 50 km. We plot the mean and standard deviation of the generated samples. For the GMM predictions, the LA21 model requires non-ergodic median site and earthquake source adjustment terms in addition to the backbone BA18 model calculation [23]. We calculate the non-ergodic logarithmic mean (μ_{mean}) of ground motion estimates, along with the mean of the standard deviation predictions.

We also show the comparison between the pre-trained and fine-tuned versions of our CGM-GM model. We found that the fine-tuning strategy can effectively improve the mean predictions and reduce the variations of FAS values under large-magnitude scenarios.

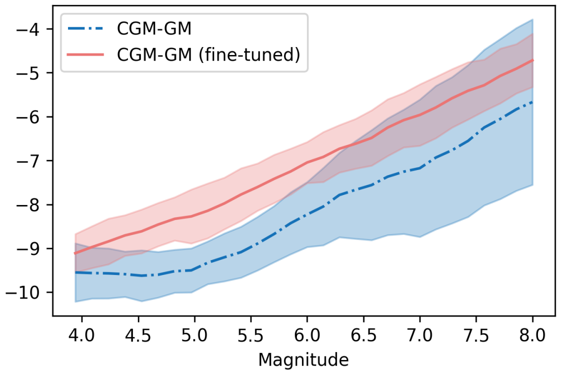

Supplementary Figure 15: Comparison of the magnitude scaling performance between our pre-trained CGM-GM model and the fine-tuned CGM-GM model over rupture distances ranging from 10 to 50 km. The solid line and the shaded area denote the mean curves and the region of mean ± std.

2.11 Spatial correlations

In this part, we provide additional supporting results regarding the learned spatial continuity from three perspectives: (i) analysis of median FAS maps at different frequencies, (ii) evaluation of semi-variograms of the predicted ground motions, and (iii) a sensitivity test using a modified earthquake scenario with a slightly perturbed source location. In our study, the spatial continuity is achieved by conditioning the model on continuous geographic coordinates.

First, all FAS maps presented in this study are computed as the medians of 30 realizations, providing a more stable and representative summary of the model output. In particular, the FAS maps at 2 Hz, 5 Hz, and 10 Hz shown in Supplementary Figures 8, 7, and Figure 2 in the main text exhibit clear spatial coherence.

Second, in addition to the visual inspection, we compute the semi-variogram [24] of the prediction as a function of separation distance (ΔS) between the predicted stations, which is another direction for measuring spatial correlations between ground motions. As shown in Supplementary Figure 16, we observe that at very short separation distances (e.g., $[0, 5]$ km), the semi-variogram values are very close to 0, meaning that the correlations of the predicted values between nearby stations are close to 1, which indicates that there is spatial continuity in the predictions across station locations. Moreover, we provide an analytical kernel function fitted to the empirical semivariogram, expressed as

$$\gamma(\Delta S) = 1 - \exp\left(-\frac{b \cdot \Delta S^2}{2}\right). \tag{10}$$

Here, $\gamma(\cdot)$ denotes the semi-variogram function, and b is a fitting parameter in the kernel function. The estimated values of b for the 2 Hz, 5 Hz, and 10 Hz cases are 4.858×10^-3, 4.033×10^-3, and 3.86×10^-3, respectively. These correspond to correlation lengths of $\rho = 14.35$, 15.75, and 16.10 km, respectively. The correlation length defines the spatial distance beyond which the correlation between data points significantly decreases. A correlation length of approximately 15 km is reasonable, rejecting the assumption of white noise. These findings imply the presence of spatial structure and provide further quantitative evidence supporting the spatial continuity of the generated ground motions.

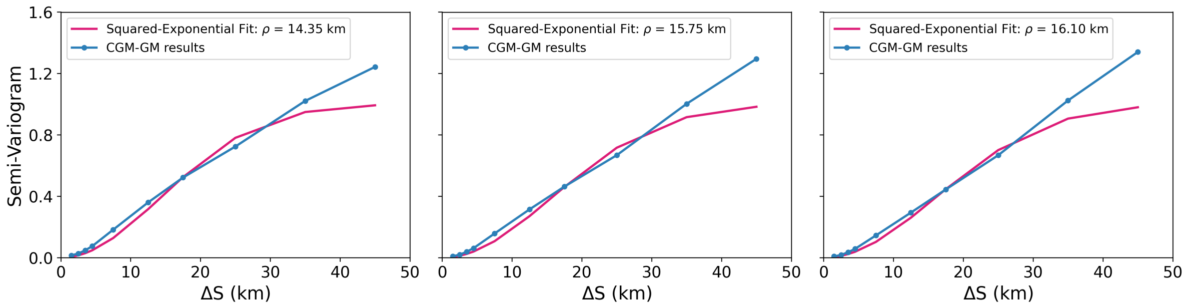

Supplementary Figure 16: The empirical correlations at 2 Hz (left), 5 Hz (mid), and 10 Hz (right).

To further demonstrate spatial continuity between predictions across event locations, we slightly move the coordinates of the event location and compute new FAS maps. Specifically, the original seismic event is characterized by a magnitude of 3.84 and a depth of 7.94 km. The epicenter, denoted by a red star, is located at a geographic position with a latitude of $37°51.6'N$ and a longitude of $122°15.6'W$. We modify the source location 1.4 km South while keeping all other physical parameters unchanged. The comparison between the new and original FAS maps at 10 Hz is shown in Supplementary Figure 17. The resulting map exhibits a good similarity to the original,

further demonstrating that our CGM-GM model maintains spatial continuity in its predictions across different event locations.

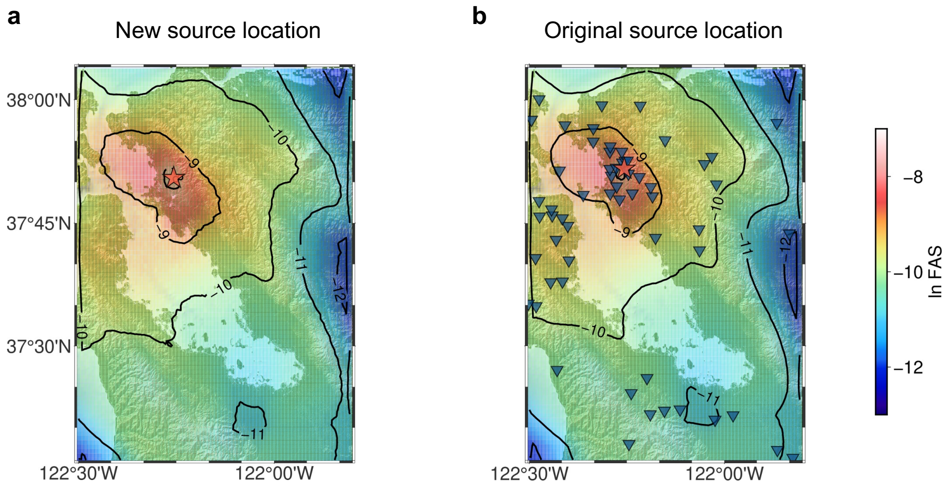

Supplementary Figure 17: The predicted Fourier Amplitude Spectra (FAS) maps (10 Hz) with new and original source locations from our CGM-GM model. The results are both based on the median FAS values from 30 realizations.

References

[1] Diederik P. Kingma and Max Welling. Auto-encoding variational bayes. In *International Conference on Learning Representations*, 2014.

[2] Danilo Jimenez Rezende, Shakir Mohamed, and Daan Wierstra. Stochastic backpropagation and approximate inference in deep generative models. In *International Conference on Machine Learning*, pages 1278–1286. PMLR, 2014.

[3] Ian Goodfellow, Yoshua Bengio, and Aaron Courville. *Deep learning*. MIT press, 2016.

[4] Geoffrey E Hinton and Ruslan R Salakhutdinov. Reducing the dimensionality of data with neural networks. *Science*, 313(5786):504–507, 2006.

[5] Diederik P Kingma, Max Welling, et al. An introduction to variational autoencoders. *Foundations and Trends® in Machine Learning*, 12(4):307–392, 2019.

[6] Laurent Girin, Simon Leglaive, Xiaoyu Bie, Julien Diard, Thomas Hueber, and Xavier Alameda-Pineda. Dynamical variational autoencoders: a comprehensive review. *Foundations and Trends in Machine Learning*, 15(1-2):1–175, 2022.

[7] Diederik P. Kingma and Jimmy Ba. Adam: a method for stochastic optimization. In *International Conference on Learning Representations*, 2015.

[8] Ian Goodfellow, Jean Pouget-Abadie, Mehdi Mirza, Bing Xu, David Warde-Farley, Sherjil Ozair, Aaron Courville, and Yoshua Bengio. Generative adversarial nets. In *Conference on Neural Information Processing Systems*, volume 27, 2014.

[9] Jascha Sohl-Dickstein, Eric Weiss, Niru Maheswaranathan, and Surya Ganguli. Deep unsupervised learning using nonequilibrium thermodynamics. In *International Conference on Machine Learning*, pages 2256–2265. Proceedings of Machine Learning Research, 2015.

[10] Jonathan Ho, Ajay Jain, and Pieter Abbeel. Denoising diffusion probabilistic models. In *Conference on Neural Information Processing Systems*, volume 33, pages 6840–6851, 2020.

[11] Sam Bond-Taylor, Adam Leach, Yang Long, and Chris G Willcocks. Deep generative modelling: a comparative review of vaes, gans, normalizing flows, energy-based and autoregressive models. *IEEE Transactions on Pattern Analysis and Machine Intelligence*, 44(11):7327–7347, 2021.

[12] Zhengfa Bi, Nori Nakata, Rie Nakata, Pu Ren, Xinming Wu, and Michael W Mahoney. Advancing data-driven broadband seismic wavefield simulation with multiconditional diffusion model. *IEEE Transactions on Geoscience and Remote Sensing*, 63:1–9, 2025.

[13] Martin Arjovsky, Soumith Chintala, and Léon Bottou. Wasserstein generative adversarial networks. In *International Conference on Machine Learning*, pages 214–223. Proceedings of Machine Learning Research, 2017.

[14] Thomas Lucas, Konstantin Shmelkov, Karteek Alahari, Cordelia Schmid, and Jakob Verbeek. Adaptive density estimation for generative models. In *Conference on Neural Information Processing Systems*, volume 32, 2019.

[15] Cheng Lu, Yuhao Zhou, Fan Bao, Jianfei Chen, Chongxuan Li, and Jun Zhu. Dpm-solver: a fast ode solver for diffusion probabilistic model sampling in around 10 steps. In *Conference on Neural Information Processing Systems*, volume 35, pages 5775–5787, 2022.

[16] Tariq Anwar Aquib and P Martin Mai. Broadband ground-motion simulations with machine-learning-based high-frequency waves from fourier neural operators. *Bulletin of the Seismological Society of America*, 114(6):2846–2868, 2024.

[17] John G Anderson. Quantitative measure of the goodness-of-fit of synthetic seismograms. In *Proceedings of the 13th World Conference on Earthquake Engineering*, volume 243, page 243. Earthquake Engineering Research Institute, 2004.

[18] Kim B Olsen and John E Mayhew. Goodness-of-fit criteria for broadband synthetic seismograms, with application to the 2008 mw 5.4 chino hills, california, earthquake. *Seismological Research Letters*, 81(5):715–723, 2010.

[19] Katsuaki Konno and Tatsuo Ohmachi. Ground-motion characteristics estimated from spectral ratio between horizontal and vertical components of microtremor. *Bulletin of the Seismological Society of America*, 88(1):228–241, 02 1998.

[20] Grigorios Lavrentiadis, Norman A. Abrahamson, and Nicolas M. Kuehn. A non-ergodic effective amplitude ground-motion model for california. *Bulletin of Earthquake Engineering*, pages 1–32, 2021.

[21] Evan Hirakawa and Brad Aagaard. Evaluation and updates for the USGS San Francisco bay region 3D seismic velocity model in the east and north bay portions. *Bulletin of the Seismological Society of America*, 2022.

[22] Mark Leonard. Earthquake fault scaling: self-consistent relating of rupture length, width, average displacement, and moment release. *Bulletin of the Seismological Society of America*, 100(5A):1971–1988, 2010.

[23] Jeff Bayless and Norman A. Abrahamson. Summary of the ba18 ground-motion model for fourier amplitude spectra for crustal earthquakes in california. *Bulletin of the Seismological Society of America*, 109(5):2088–2105, 09 2019.

[24] Nirmal Jayaram and Jack W Baker. Correlation model for spatially distributed ground-motion intensities. *Earthquake Engineering & Structural Dynamics*, 38(15):1687–1708, 2009.
